# An Enhanced Posture Prediction-Bayesian Network Algorithm for Sleep Posture Recognition in Wireless Body Area Networks

**DOI:** 10.1155/2022/3102545

**Published:** 2022-05-28

**Authors:** A. Roshini, K. V. D. Kiran

**Affiliations:** Department of Computer Science and Engineering, Koneru Lakshmaiah Education Foundation, Vaddeswaram, AP, India

## Abstract

Wireless body area networks have taken their unique recognition in providing consistent facilities in health monitoring. Several studies influence physiological signal monitoring through a centralized approach using star topology in regular activities like standing, walking, sitting, and running which are considered active postures. Unlike regular activities like walking, standing, sitting, and running, the in-bed sleep posture monitoring of a subject is highly necessary for those who have undergone surgery, victims of breathing problems, and victims of COVID-19 for whom oxygen imbalance is a major issue as the mortality rate in sleep is high due to unattended patients. Suggestions from the medical field state that the patients with the above-mentioned issues are highly suggested to follow the prone sleep posture that enables them to maintain the oxygen level in the human body. A distributed model of communication is used where mesh topology is used for the data packets to be carried in a relay fashion to the sink. Heartbeat rate (HBR) and image monitoring of the subject during sleep are closely monitored and taken as input to the proposed posture prediction-Bayesian network (PP-BN) to predict the consecutive postures to increase the accuracy rate of posture recognition. The accuracy rate of the model outperforms the existing classification and prediction algorithms which take the cleaned dataset as input for better prediction results.

## 1. Introduction

Sleep posture analysis has taken its significance in health monitoring, where the health status of patients with mental and physical disorders can be under surveillance. In-bed postures during sleep assess the sleep quality that could be used for medical diagnosis. Sleep recognition focuses on three levels: sleep position recognition, sleep stage recognition, and insomnia detection, including gathering and analysis of multisensor sleep data.

Force-sensitive application (FSA) pressure mapping mattresses containing 2D—an array of sensors—are used to classify the sleep postures of the subject to test the accuracy of the algorithm [1]. However, this model seems expensive and does not consider the prone posture. A neural network classifier is used for position classification with a piezoelectric sensor and pressure sensor, followed by consecutive position identification by a Bayesian classifier. The accuracy % of the postures shows that with the optimized number of sensors, better results can be yielded [2].

A classification model of sleep stage analysis based on electrocardiogram (ECG) signal using particle swarm optimization (PSO) and weighted extreme machine learning (WELM) for feature selection and to deal with imbalanced learning dataset was used [3]. The technique focuses on nonrapid eye movement and wake stage for sleep stage classification.A model that addresses three levels of sleep recognition, like sleep posture, sleep stage recognition, and insomnia detection using a self-supervised sleep recognition model (SSKM) with two different pressure sensing mats, was adopted [4].

The accuracy rate of sleep position classification using a convolutional neural network (CNN) yields 94.0%, outperforming the other classification algorithms [5]. IoT enabled a sleep posture recognition system which uses CNN for classifying the posture to trigger full warning, reduce pressure sores and unoccupied bed alerts, and pane 90% of accuracy in sleep posture classification [6]. A traditional neural network for position classification with extension to position transition restriction using a Bayesian network also improves the accuracy of posture detection by reducing errors [7].

An intelligent sleep detection system with force sensual resistor sensors (FSR) to analyze the sleep position classification hardened the accuracy using the *K*-nearest neighbor algorithm with 91% [8]. ECG signals extracted from 12 capacitively coupled electrodes provide better classification among the basic lying posture. Such a system can be used for better heartbeat and sleep monitoring and with body posture detection [9]. Ultrawideband radar s/m is used for the classification of human sleep postures, where a multiview learning model sleep sent with time-series data augmentation is used for data classification [10].

In this study, an accelerometer sensor is used to acquire the heartbeat rate (HBR) that is gathered from nonintrusive sensors to predict the in-bed position classification from two sensors that are used. The prediction is made with four classes of sleep postures, and the identification of the posture is useful for patients suffering from breathing problems. The prone sleeping posture is mainly focused on as the position is suitable for maintaining better oxygen levels in the human body. In a recent article, the doctors have suggested that COVID-19 balances the oxygen levels of the victims who are under the surveillance of the medical team. The following assumptions are made in this work:

- Four sleep levels are considered: prone, supine, right lateral recumbent, and left lateral recumbent
- The posture prediction-Bayesian network (PA-BN) is used for the prediction of succeeding sleep posture (or) sleep position transition, and the convolutional neural network (CNN) is used for classification

## 2. Literature Survey

An unobtrusive sleep posture classification with blanket-enabled sensors is used with an infrared depth camera for data acquisition. The sleeping postures considered in various levels include supine, prone, log, fetal, and under four blankets, and the participants include 40 men and 26 women. Image processing and feature addition are done using affine transformation and data fusion techniques thereby enhancing the collected blanket conditions without modifying the collected data [10]. The log and fetal postures were merged into the side-lying posture, and the prone posture was pooled in coarse classification. A comparison study for analyzing the accuracy of the system through the F1 score under blankets produced a result of 89% classifying seven sleep postures.

A low-cost pressure array is embedded with conductive fabric and conductive wires, where sensors are deployed with bedspreads configured as 32 rows and 32 columns with a total of 1024 nodes. The paper considers Arduino nano for collecting data using a 10-bit analog-digital converter (ADC) [11]. The data acquisition was done considering six sleeping postures from five participants, with the convolutional neural networks implemented on a PC. The accuracy rate of the model yields 84.80% with the standard training test method and 91.24% with learning-based subject-specific methods.

Health monitoring of an elderly person is very important during bedtime, as when the body is under rest, the variation in the physiological signal is high. In addition, during sleep, timeless attention is given by the caregivers, which affects the sleep quality leading to health issues [12]. The model considers three sleep postures for analysis. A novel sleep detection system (SDS) uses Flexi Force Sensors (FFS), which are a resistive-based technology producing analog signals for the data gathered. The accuracy rate of sleep posture classification follows the random forest method with 90%.

The sleep posture classification has taken its advancement in sleep posture recognition with a matching-based approach termed as Body-Earth mover's distance. The similarity level of a posture is identified using weighted 2-dimensional shapes combined with the earth mover's distance and Euclidean distance for estimation of the similarity index [13]. Neither multiple features nor unobtrusive sleep posture is collected to recognize the sleep posture. The study analyzes six different sleeping postures for around 14 persons. The method outperforms the existing methodologies with an accuracy rate of 91.21%.

An algorithmic framework for sleep posture recognition to avoid pressure ulcers is done using a smart mat system based on a dense, flexible sensor array and printed electrodes. The model considers a dense, flexible sensor array for providing high comfort and high resolution for long-time sensing. Preprocessing of the acquired data is done using deep residual networks [14]. The advantage of using such a system is that the complexity of the feature extraction process can be reduced. The work considers four sleep stages experimented on seventeen subjects yielding an accuracy rate of 95.08%.

Camera-based vision methods are used to estimate of the in-bed pose with a notable difference in lighting conditions on a complete day by considering different postures during sleep, rather than considering a conventional neural network, a pretrained convolution neural network model that takes public datasets of human in-bed postures. The infrared selective image acquisition technique (IRS) retains the quality of the data collected under different lighting conditions [15]. The errors in the trained dataset are rectified using the histogram of the oriented gradient method. The intermediate layers of the neural network are fine-tuned and increase the accuracy rate of posture estimation by 26.4% from the existing methods.

A multimodal support vector machine (SVM) technique is used for sleep posture classification. The posture patterns are categorized by using both a pressure sensor map and a video image. The multimodal sensing technique considers joint feature extraction and data fusion as two parameters for the classification of the patterns that are acquired [16]. The multimodal approach outperforms the single modal sensing as a broad classification is performed in this approach, and the accuracy rate is also increased to a certain level.

The Bayesian network with a belief propagation model estimates the sleep postures by deploying the rigid link model of the human body that enables tracking the motion of the subject [17]. The estimation of postures follows in three dimensional using a probabilistic inference way, which utilizes 15 nodes at the joints connected by rigid links. Bayesian probabilistic inference is interpreted using belief propagation. The validation of the model is done using the Bayesian network that takes a graphic network as input, and the results show that the method outperforms with better accuracy in posture estimation. The limitation in the proposed model lies in the data that are considered, which are synthetic.

An integration model combining extreme learning machine (ELM) and particle swarm optimization (PSO) is used for selecting features and determining the hidden nodes. The paper also highlights how a portable sensor device is used to track the physiological signal to provide continuous health care monitoring of a patient. A model demonstrates the sleep stage classification by taking the heart rate variability extracted from the electrocardiogram (ECG) [18]. A comparative study showing how the proposed system surmounts the support vector machine is analyzed. The experimental results show that better accuracy is attained with the PSO technique with a limited class.

A dense pressure-sensitive bed sheet that captures 32 features extracted from the pressure sensors uses comfortable textile sensors that produce high-resolution pressure sensors. The posture recognition classifiers use novel frameworks for pressure image analysis to characterize the different sleep postures. Three heuristics for sparse classification used in these experiments use the maximum sum of class coefficients and minimum class residual heuristics for reliable sleep posture recognition. The accuracy rate of the proposed model yields an accuracy rate of 78% with a minimum number of features.

An accurate model for sleep stage classification takes the heart rate variability (HRV) extracted from an electrocardiogram (ECG). The quality of sleep is accessed using the sleep stages that a subject exhibits. Hidden node detection of sleep features is calculated using extreme learning machine and public swarm optimization (PSO) [19]. The results are then compared with the existing classification methods like support vector machine (SVM) and ELM, but the comparative analysis shows that the accuracy rate of the existing system provides better results with varying classes of postures.

The existing system makes use of pressure sensor mats that consist of pressure sensor arrays that are embedded in the mattress on which the patient rests, which is composed of more than 1024 sensors. Support vector machines (SVM) classify the sleep postures with 1768 uniformly distributed pressure sensors with an accuracy rate of 77.14 to enhance the existing work [20], and the minimum class residual classifier uses 8192 force sensing resistors (FSR) that categorize six sleep postures with the accuracy rate of 83.02% [21]. Similarly, other classifiers like deep neural networks, *K*-nearest neighborhood, fully connected networks, and EMD yield better results in classifying the sleep postures. But the existing system considers a mesh network where the sensors are placed in five key positions to gather the heartbeat rate. The difference in considering the nonpressure sensors is to avoid the false values that are acquired from the sensors.

### 2.1. Effect of Sleep Postures on Human Health

Sleep positions have a significant influence on health conditions, age, and BMI rates. People suffering from cardiac issues and breathing problems and patients who have undergone surgery exhibit high variations as they might experience discomfort in certain sleeping positions. A study conducted by the Danish Physical Activity Cohort with Objective Measurements (DPHACTO) using the accelerometers observed that participants spent an average of 54.1% sleeping in the left/right lateral recumbent position, 37.5% in the prone position, and 7.3% in the supine position [21]. A proportional reduction in the sleep positions is observed for prone and supine cases compared with RLR and LLR as the comfort during sleep increases peaceful sleep. For every hour of observation, an average of 1.6 positions shifts per hour. It was observed that females exhibited fewer shifts when compared to male, and extended to it, people belonging to age groups 20-34 years had more hand, thigh, and back movements.

### 2.2. Questionnaire Data

**Figure 1:**
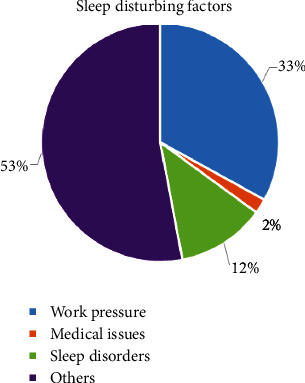
Survey report on sleep disturbance.

A survey was taken from a group of people whose age range is from 16 to 75 years, but the major participants fall under the age group 16-30 years during the second wave of COVID-19 focusing mainly on sleep time, and the obstacles that disturb the sleep during nights are shown in Figure 1. The report of the survey depicts that the time the subject goes to sleep and the activities in bed have a difference of 2 hours where most of them spend time mobile surfing, taking care of the kids, reading, etc. In addition to the time to sleep issues, the main factors that disturbed the sleep among the participants were work pressure, medical issues, sleep disorders, and other issues. The final observation was made on the type of employment where people in private work are facing major issues compared to those who are working in government organizations.

## 3. System Design and Implementation

### 3.1. System Design

**Figure 2:**
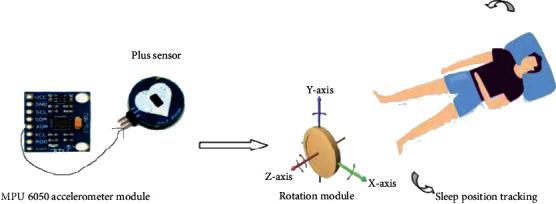
System design.

The proposed work considers a unique embedded system model to receive the physiological sensors that comprise an accelerometer sensor and a Wi-Fi chip to transmit the data to the sink node. An MPU 6050 with a 3-axis accelerometer sensor and digital motion processor with an on-chip pulse sensor shown in Figure 2 accesses a local network, which is used to continuously monitor the heartbeat rate. The sensor nodes placed in the chest and knee acquire the signal when the gyros are rotated, which is then amplified, demodulated, and filtered to produce a voltage that is proportional to the angular rate [22]. The 16-bit ADC digitizes the voltage at each axis. The accelerometer with microelectromechanical (MEMS) technology is used to detect the angle of tilt or inclination along the *x*-, *y*-, and *z*-axes related to the sleep postures exhibited during sleep.

### 3.2. Pulse Sensor

The heartbeat sensor connected to the MPU board consists of an LED and a light detecting resistor. The variation caused by the heartbeat influences the blood flow where the light reflected by the blood is received by the light detector. The density of the light (*L*) absorbed or reflected depends on the blood volume in that tissue, which is proportional to the heartbeat rate.

(1) $$\begin{array}{c} L\;\alpha \text{ HBR}. \end{array}$$

### 3.3. Sleep Posture Synthesis

Sleep postures corresponding to the heartbeat rate extracted from the accelerometer pulse sensor are normalized by ruling out the environmental noise and the physiological features exhibited by the human body. Threshold filtering and Gaussian filtering normalization techniques are used to process the sleep postures captured.

### 3.4. Threshold Filtering

The sleep posture images are filtered based on the pulse sensor array, which is 64 × 64. These values are used as the threshold points for normalizing. The pulse point of the image is considered as *H*_*a*,*b*_, where 1 ≤ *a* ≤ 64 and 1 ≤ *b* ≤ 64. The threshold value is now calculated as

(2) $$\begin{array}{c} \text{PulThr}=\frac{\sum_{a=1}^{x}Ha\sum_{b=1}^{y}Ha}{H^{\prime }}, \end{array}$$

where *H*′ is the number of void pulse points. If the pulse point is considered null for the least threshold values,

(3) $$\begin{array}{c} H_{a,b}=\left\{\begin{array}{c} H_{a,b},\;\text{if }H_{a,b}\leq \text{PulThr},\\ 0,\;\text{ otherwise}. \end{array}\right. \end{array}$$

### 3.5. Gaussian Filtering

The probability distribution for the noise removal in the images is derived using the Gaussian filtering method. A 2D Gaussian function is used to convolve the distribution function with the image. A symmetric 5 × 5 unit is applied to remove the noise from the images. The sampling rate of the 2D Gaussian kernel coefficient is given by

(4) $$\begin{array}{c} G\left(x,y\right)=\frac{1}{2\pi \sigma^{2}}e^{-\left(\;x^{2}+y^{2}\right)/2\sigma 2}. \end{array}$$

### 3.6. Rotation Principal Component Analysis

Unlike the above two [13] preprocessing procedures used to normalize the image dataset from MPII and the dataset captured from the subjects considered for the experiment, the heartbeat rate corresponding to the movement of the human body adopts principal component analysis that will rotate the accelerometer sensor in order to capture the variations during sleep. The angle of rotation is given by the leading eigenvector of the PCA and the protracted edge of the sleep image.

Principal components of the data variables that are orthogonal to each other are determined. The heartbeat signal received is considered a 2D matrix, *X*_*i*,*j*_ with dimensions *N* × *M*, where *i* = 1, 2, ⋯, *N* and *j* = 1, 2, ⋯, *M* denote the fast and slow time indices, respectively. The variables *i* and *j* represent the index of variables and the index of observations for PCA. A simple, consistent physiological processing methodology is applied to enhance the signal quality by applying the below logic:

(5) $$\begin{array}{c} X_{\left(i,j\right)}=\frac{1}{2R+1}\;\overset{j+R}{\underset{l=j-R}{\sum }}X\left(i,j\right). \end{array}$$

The following steps are followed to normalize the larger database into a set of summary indices for better visualization:

The mean-centered value is calculated by subtracting with the matrix *X*:

(6) $$\begin{array}{c} X=X-\frac{1}{M}\;\overset{M}{\underset{j=1}{\sum }}X\left(i,j\right). \end{array}$$

The covariance matrix is computed, followed by the eigenvalues (*λ*) and eigenvectors (∧) for eigen vectors some irrelevant variables are seen,

(7) $$\begin{array}{c} C\Lambda =\lambda \;\Delta ,\\ C={\text{USV}}^{T}. \end{array}$$

Finally, the projection of matrix *X* mapping onto the *k*^th^ eigenvector ∧_*k*_ is generated.

(8) $$\begin{array}{c} Y_{k}=U_{k}^{T}X. \end{array}$$

## 4. Data Acquisition and Processing

The data required for this work consists of three parts namely: (a) numerous images of human beings from the MPII dataset, (b) the video clips captured in sleep postures, and (c) the heartbeat rate signal extracted from the accelerometer sensors implanted in the human body. The MPII dataset is a collection of YouTube clips cropped that is based on human posture variations [23]. Image corner positions, including human bodies, were labeled by the MPHB dataset. The images were then reshaped into 32 × 32 resolution, which was labeled as “11.” The nonhuman images that are ruled out were labeled as “00.” A collection of these 7000 images was converted to grayscale and normalized to a range of 0 and 1. The image data utilized for the training model were stored as 32-bit float points on the hard disk.

The experimental setup for image acquisition is demonstrated using a thermal imager with a resolution of 640 × 480, and a line pixel of 17 *μ*m is set up to prevent external vibrations and noise in the experimental procedure. The range of the color wavelengths and the temperature differences are 7.5 ± 14 *μ*m and 0.5ÊC, respectively [20]. An infrared camera is equilibrated on top of the thermal image such that the field of view is uniform. The acquired data is then transferred to the server with the USB and patch cables. Customized image gathering software is used for the generation of both thermal and infrared videos. The video clips are then fed into MATLAB R2020 for further analysis of the consecutive sleep postures. The workflow of the infrared lamps is controlled using photosensitive sensors. The image capturing process is managed during the nighttime with an IR-cut filter embedded in the IR camera.

The heartbeat dataset was used as the training data collected from 3 subjects for various postures like sleep, stand, sit, run, forward bend, and backward bend by attaching the pulse sensor and accelerometer sensors to the subject's right wrist, abdomen, right thigh, left thigh, and left shoulders [22]. A set of 5 pairs of 3-axis acceleration in the *x*, *y*, and *z* directions extract data from the 5 sensors is transmitted using a NodeMCU to the central server from which data is taken to the PC. A total of 44,800 samples were collected from the experimental setup, but in this paper, the data samples corresponding to the sleep postures are considered the input that will be taken for training the model.

### 4.1. Histogram of Oriented Gradients

To normalize the space, cost, and weight for meaningful extraction of sleep posture images, the histogram of gradients normalization method is used. This method is an efficient way to extract the objects for object detection that removes the offset of weights, position, and size of different objects. The filters are applied in horizontal and vertical directions by predefining the size of the cells and the number of cells in one block [11]. This work focuses on the weight and space normalization of images.

The weight normalization is based on the pressure points which are given by

(9) $$\begin{array}{c} {\text{HOG}}_{\left(W\right)}=\frac{\text{Pressure Point}}{\sum_{i=1}^{n}\text{I}\text{nitial Pressure points}},\\ Sf={\left(\frac{Wn}{W}\right)}^{1/2}. \end{array}$$

### 4.2. Posture Prediction Model

**Figure 3:**
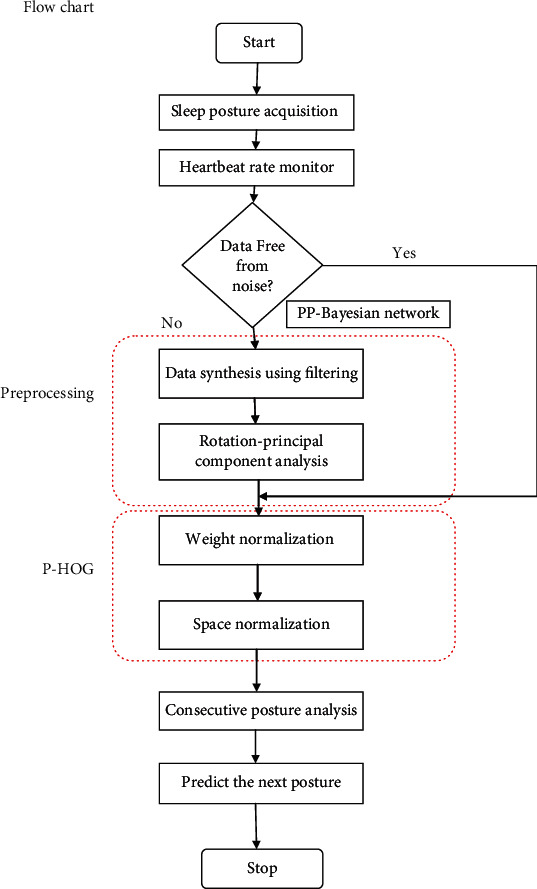
PP-BN process flow.

The prediction of consecutive postures is accessed from the video dataset captured using far-infrared images and an infrared camera enabled with a cut lens and infrared lighting array. A sequence of image frames numbered with continuous index values is stored for training the unsupervised model. These frames are then correlated with the heartbeat rate. The probe and supine posture variants are considered for training purposes [24]. Followed by training, the Bayesian network prediction is included through which the current sleep position is based on the previous position exhibited by the subject, as shown in Figure 3. The image frames derived are of size 64 × 64-pixel resolution for better prediction and image identification under critical situations.

## 5. Methodology

### 5.1. Prediction Using Posture Prediction-Bayesian Network (PP-BN)

The classification of sleep posture is preceded by the prediction of different in-bed positions exhibited by a subject during sleep. A probabilistic graphical model that represents the four sleep postures considered in this work was used: supine, prone, RLR, and LLR follow the Bayesian network to estimate the next possible sleep posture from the current in-bed position. This posture prediction model turns down the noise signals that are captured along with the physiological data due to [7] the uncontrolled environment setup of the devices used or due to the probability of the sequential posture prediction which is estimated from the preceding position and the current physiological data extracted.

(10) $$\begin{array}{c} P\left(A,B\right)=P\left(A\right)–P\left(\frac{A}{B}\right),\\ P\left(\frac{A}{B}\right)=P\left(\frac{P_{n}}{P_{n-1}},P_{n-2}\right)-P\left(\frac{A}{B_{i}}\right), \end{array}$$

where *P*_*n*_, *P*_*n*−1_, and *P*_*n*−2_ are the position captured at (*n* − 2)^th^, (*n* − 1)^th^, and *n*^th^ time position of the posture sequence.

### 5.2. Transition Model for Sleep Posture Prediction

**Figure 4:**
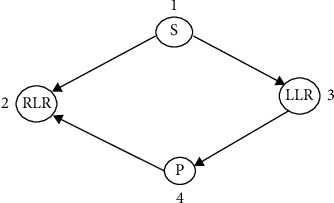
PP-BN model.

The PP-Bayesian network takes the raw data that are acquired from the sensors that are implanted on the wrist, shoulders, thighs, knee, and abdomen. The transition model labeled in Figure 4 shows the transition model that will depict the sleep model.

The gathered data includes the physiological signal extracted from the pulse sensor and the video signal. The Bayesian network model is used to evaluate the environmental factors affecting the signal. The datasets considered in this article are briefed in the data acquisition section.

The following assumptions are made before posture prediction is carried out.

1. The initial position is on the bed
2. The prone position is attained only after the LLR or RLR
3. LLR or RLR is attained only after supine position

The joint probability distribution that can be computed based on the above assumptions returns the relationship between two variables. The normalized signal and image data acquired are classified into three levels for accuracy calculation: low, moderate, and high by transforming the continuous values of the signal values to par values.

**Figure 5:**
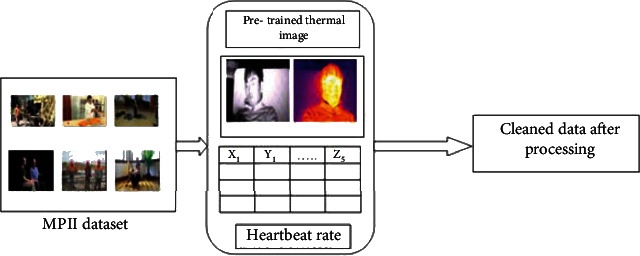
Data synthesis of the sleep dataset.

The process of synthesis includes the heartbeat rate extracted during the sleep posture, followed by determining the image frames from the video clips captured, after which the image relevancy from the image frames and MPII dataset is compared as a trained image and subset of the MPII dataset called as the sleep subset as shown in Figure 5. The image pixels are visualized as a matrix integrating the physiological signals to remove the redundancy or process the uncleaned dataset collected directly from the environment. The results shown in this work yields better accuracy as the cleaned dataset is taken as an input for predicting as in Algorithm 1 the successive posture. This is the main advantage for remote health monitoring and telemedicine system for handling the patients and managing the critical data extracted from the human body. The model has limited its scope to adults and those who have undergone surgeries and require an external caretaker.

## 6. Experiment and Results

**Figure 6:**
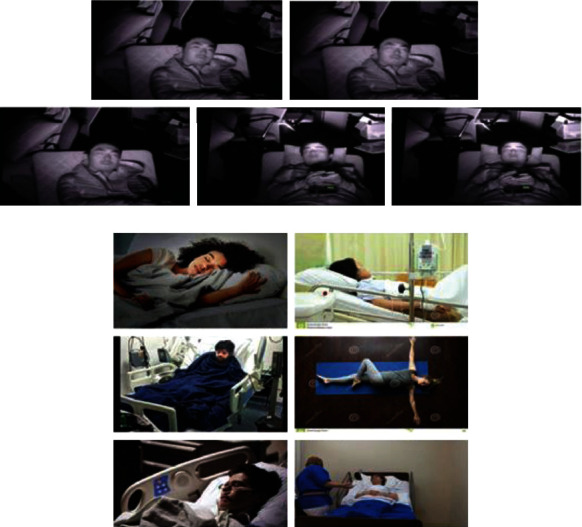
(a) Extraction of image frames from the sleep video. (b) The subset of the MPII dataset based on the extracted sleep posture.

The dataset considered in this work is an outcome of the experiment carried out that includes both male and female gendered subjects of age varying from 25 to 60 years. The BMI of the subjects falls between 55 and 85 kilograms, and their height is between 155 and 185 centimeters. The dataset is a collection of sleep videos using infrared cameras monitoring both the breathing rate and heartbeat rate. The trained model considers six sleep postures, as shown in Figure 6(a), which are integrated with the heartbeat rate and given as input to the prediction system. Although the system should provide the result for the six postures, the system was tested only on the supine posture.

The prediction model's result includes the following steps: (a) conversion of the videos into image frames and generating the subset of the MPII dataset as shown in Figure 6, (b) processing of the frames to remove the environmental factors using threshold filtering with the threshold value set to 0.5725 based on the intensity of the image, and (c) the Bayesian prediction model then mapping the heartbeat rate and the sleep postures for the prediction of the next consecutive sleep positions for patients' health monitoring.

**Figure 7:**
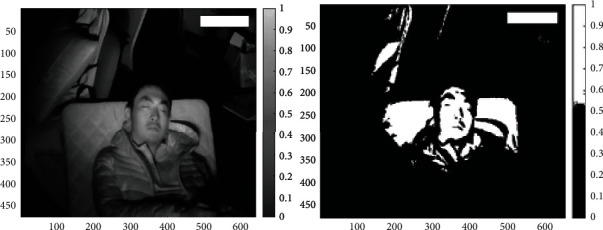
Threshold filtering.

**Figure 8:**
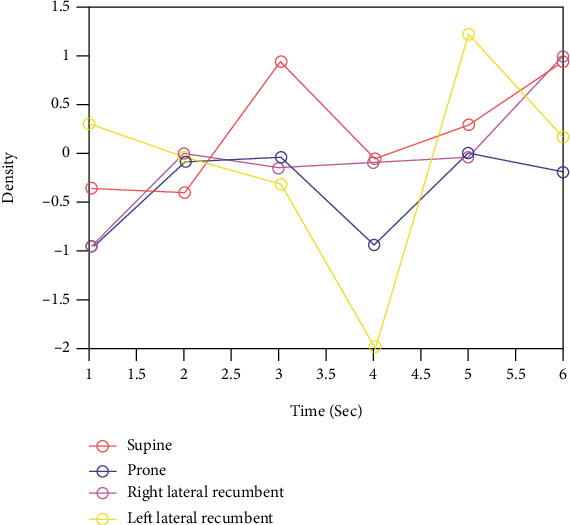
Sleep posture prediction rate.

The filtering techniques and preprocessing steps provide better performance and improve the accuracy of the prediction model when compared to the previous work; the proposed results consider the threshold filtering as shown in Figure 7, Gaussian filtering, and rotation principal component analysis followed by a histogram of gradients classification for better posture identification; the existing method does not consider the feature-based approach that depends on local features and is absolutely more sensitive to noises than the semantic matching approaches [25]. This accuracy is a result of two factors: (1) histograms derived from global descriptors that will better describe the symmetric character of supine and prone postures and (2) increasing the robustness of the model by considering all postures from the dataset.  Figure 8 shows that increase in density of the image improves the posture prediction rate, and out of the four postures considered, all four postures show relative accuracy improvement in predicting the posture.

## 7. Discussion

The comparative study shown in Table 1 considers various datasets by which the performance varies in terms of accuracy. Hence, the comparison is mainly focused on the postures considered in the initial works considering the off-bed, sitting, lying center, lying left, and lying right, whereby the prone posture is not considered in deep neural networks and *K*-nearest neighborhood algorithm [11]. The next important comparison parameter is the number of sensors used. Although the accuracy rate of the former algorithms is high, the usage of sensors used makes the model highly expensive. This proposed work derives the required accuracy rate by covering the most frequent postures exhibited during sleep.

## 8. Conclusion

A collective approach that considers the sleep videos followed by preprocessing of the extracted frames using filtering and principal component analysis method integrated with heartbeat rate has outperformed the accuracy rate by 0.90% when compared with the previous system. The system mainly focuses on triggering caretakers of patients under postsurgery remote health monitoring and will be a remedy for the COVID victims having breathing issues. The sleep analysis study estimates the intensity of the image to predict the successive postures and the variations in the physiological signals.

## 9. Future Work

The technique shows that the channelization problems in the MAC layer hinder the data gathering process and affect the accuracy rate of the extracted signal for health monitoring. The PA-BN protocol is used to acquire the physiological data followed by classifying the patterns for various sleep positions, and the model can be extended by applying machine learning algorithms and classification strategies for other physiological signals.

## Figures and Tables

**Algorithm 1:**
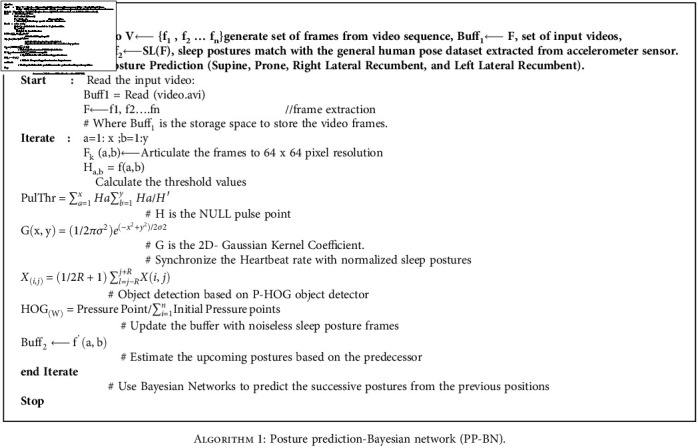
Posture prediction-Bayesian network (PP-BN).

**Table 1:** Comparative chart of sleep posture recognition.

S. no	Sensor name	No. of subjects	No. of sensors	Algorithm	Accuracy rate	Year of work
1	Uniformly distributed pressure sensors	10	1768	Support vector machines	77.14%	2015
2	Uniformly distributed FSR sensor	19	3200	Deep neural networks	99.70%	2018
3	Matrix of FSR sensors	6	NA	Template matching by a minimum mean squared error	96.10%	2017
4	Uniformly distributed pressure sensors	12	1728	Fully connected networks	97.90%	2020
5	Uniformly distributed pressure sensors	19	3200	Deep neural networks	97.10%	2018
6	Uniformly distributed pressure sensors	14	8192	EMD+*K*-nearest neighborhood	91.21%	2016
7	Uniformly distributed pressure sensors	16	1024	ResNet	90.08%	2021
8	Accelerometer pulse sensor (our method)	14	5	Posture prediction-Bayesian network (PP-BN)	91.05%	2022

## Data Availability

The data are available at https://drive.google.com/drive/folders/1HsKQeAa26CrujkJeVH-gu1j22OwioXwe?usp=sharing.
